# Carnosine Supplementation Has No Effect on Inflammatory Markers in Adults with Prediabetes and Type 2 Diabetes: A Randomised Controlled Trial

**DOI:** 10.3390/nu16223900

**Published:** 2024-11-15

**Authors:** Saeede Saadati, Maximilian de Courten, Cyril Deceneux, Magdalena Plebanski, David Scott, Jakub Mesinovic, Paul Jansons, Giancarlo Aldini, James Cameron, Jack Feehan, Aya Mousa, Barbora de Courten

**Affiliations:** 1 Monash Centre for Health Research and Implementation (MCHRI), Faculty of Medicine, Nursing and Health Sciences, Monash University, Clayton, VIC 3168, Australia; saeede.saadati@monash.edu (S.S.); aya.mousa@monash.edu (A.M.); 2 Australian Health Policy Collaboration, Institute for Health and Sport (IHES), Victoria University, Melbourne, VIC 8001, Australia; maximilian.decourten@vu.edu.au; 3 Cancer Aging and Vaccine Laboratory, School of Health and Biomedical Sciences, RMIT University, Bundoora, VIC 3083, Australia; cyril.deceneux@rmit.edu.au (C.D.); magdalena.plebanski@rmit.edu.au (M.P.); 4 Institute for Physical Activity and Nutrition (IPAN), School of Exercise and Nutrition Sciences, Deakin University, Geelong, VIC 3220, Australia; d.scott@deakin.edu.au (D.S.); jakub.mesinovic@deakin.edu.au (J.M.); paul.jansons@deakin.edu.au (P.J.); 5 Department of Pharmaceutical Sciences, University of Milan, 20133 Milan, Italy; giancarlo.aldini@unimi.it; 6 School of Clinical Sciences, Faculty of Medicine, Nursing and Health Sciences, Monash University, Clayton, VIC 3168, Australia; james.cameron@monash.edu; 7 School of Health and Biomedical Sciences, RMIT University, Bundoora, VIC 3083, Australia; jack.feehan@rmit.edu.au

**Keywords:** carnosine, type 2 diabetes, inflammation, randomised trial, insulin resistance, cytokine, prediabetes

## Abstract

Background/Objectives: In vitro studies suggest that carnosine reduces inflammation by upregulating anti-inflammatory mediators and downregulating pro-inflammatory cytokines. However, human clinical trials examining the effects of carnosine on inflammatory biomarkers are scant. We conducted a secondary analysis of a double-blind randomised controlled trial (RCT) to examine the effects of carnosine supplementation on inflammatory markers and adipokines in participants with prediabetes or well-controlled type 2 diabetes (T2D). Methods: Out of 88 participants who were recruited, 49 adults with prediabetes or well-controlled T2D (HbA1c: 6.6 ± 0.7% [mean ± SD]) who were treated with diet and/or metformin were eligible for inclusion. Participants were randomised to receive 2 g/day of carnosine or a matching placebo for 14 weeks. We measured serum concentrations of monocyte chemoattractant protein-1 (MCP-1), interleukin (IL)-6, IL-10, C-reactive protein (CRP), tumour necrosis factor-α (TNF-α), adiponectin, leptin, adipsin, serpin, and resistin levels at baseline and after 14 weeks. The trial was registered at clinicaltrials.gov (NCT02917928). Results: Forty-one participants (M = 29/F = 12) aged 53 (42.6, 59.3) years [median (IQR)] completed the trial. After 14 weeks of supplementation, changes in pro- and anti-inflammatory cytokine and adipokine levels did not differ between the carnosine and placebo groups (*p* > 0.05 for all). The results remained unchanged after adjustment for confounders including age, sex, and anthropometric measures (e.g., body fat percentage and visceral adipose tissue). Conclusions: In individuals with prediabetes and well-controlled T2D, carnosine supplementation did not result in any significant changes in inflammatory markers. Larger RCTs with longer follow-up durations are needed to evaluate whether carnosine may be beneficial in individuals with poorly controlled T2D.

## 1. Introduction

Type 2 diabetes (T2D) is one of the major causes of disability and mortality worldwide, driven by obesity as a major contributing factor [1]. Obesity, characterised by excess body fat, contributes to pancreatic β-cell dysfunction and insulin resistance, the key pathological derangements involved in the development and progression of T2D [2]. Globally, the prevalence of obesity and T2D has been consistently increasing [3]. Many individuals with obesity develop prediabetes before they are diagnosed with overt T2D [3].

Chronic inflammation is a common feature of obesity and plays an important role in driving insulin resistance and the decline in β-cell function, ultimately leading to hyperglycaemia. This hyperglycaemia, in turn, worsens inflammation and creates a self-perpetuating cycle that exacerbates the progression of diabetes and its complications [4,5,6,7]. Chronic inflammation is reflected by altered levels of inflammatory cytokines and adipokines including C-reactive protein (CRP), interleukins (IL)-1, 6, and 10, tumour necrosis factor-α (TNF-α), adiponectin, and resistin [7,8,9,10,11]. As an endocrine organ, adipose tissue secretes both immunomodulatory cytokines and adipokines [12], which are involved in lipid and glucose metabolism, inflammation, appetite regulation, and energy balance [13], contributing to the development of metabolic disorders including obesity and T2D [13]. Therefore, in addition to reducing glucose levels in individuals with prediabetes and T2D, it is imperative to examine therapeutic agents that target inflammation and oxidative stress as key drivers of disease progression [5].

Carnosine (β-alanyl-L-histidine), a member of the family of histidine-containing dipeptides (HCDs), exerts pharmacological properties including anti-inflammatory, anti-oxidative, anti-advanced glycation end-products (AGEs), and chelating properties [14,15]. In experimental studies, histidine and carnosine intake significantly suppressed IL-6 and TNF-α levels in diabetic mice [16,17,18], but carnosine had no effects on leptin or adiponectin concentrations in rats with metabolic syndrome [19]. Human studies have reported conflicting data. Our recent systematic review and meta-analyses identified nine existing RCTs examining the effects of carnosine/HCDs on inflammation markers, with the pooled analysis demonstrating that carnosine/HCDs reduced TNF-α and CRP but had no effect on adiponectin and IL-6 [20]. However, most of the nine trials focused on β-alanine, anserine, or histidine, or were in children, with only two trials examining carnosine alone in adult populations. The first was a small pilot RCT by our group [21], whereby carnosine supplementation (2 g/day, 12 weeks) normalised serum resistin levels in 22 non-diabetic overweight or obese individuals, with no changes in leptin, adipsin, adiponectin, and CRP levels [21,22]. The second was an RCT in Iran [23], which measured three inflammation markers in 44 individuals with T2D using oral medications. Carnosine supplementation (1 g/day) for 12 weeks reduced TNF-α levels, with no changes in IL-6 or IL-1β compared with placebo.

Given the scarcity of data and the inconclusive evidence to date, this study aimed to assess the effects of carnosine supplementation on a range of inflammatory markers and adipokines in individuals with prediabetes and well-controlled T2D.

## 2. Materials and Methods

### 2.1. Study Design and Participants

This 14-week randomised, parallel-group, placebo-controlled, double-blind trial recruited 88 adults with prediabetes and well-controlled T2D who were either treated with diet or metformin. Participants were eligible if they were aged 18–70 years and diagnosed with either prediabetes (defined by impaired fasting glycaemia (IFG) with a fasting blood glucose of 6.1–6.9 mmol/L and/or impaired glucose tolerance (IGT) with a 2 h blood glucose of 7.8–11.1 mmol/L) or with T2D (indicated by fasting blood glucose ≥ 7.0 mmol/L and 2 h blood glucose of ≥11.1 mmol/L) and were treated with diet or metformin only. Participants were required to have a stable body weight with no intention to lose weight or change physical activity throughout the trial. Exclusion criteria were a haemoglobin A1c (HbA1c) concentration greater than 8%, body mass index (BMI) > 40 kg/m^2^, being a current smoker or drinking alcohol (more than four standard drinks/week for men and two standard drinks/week for women), having a history of blood transfusion in the last three months, or taking dietary supplements or medications that impact cardiometabolic measures (other than metformin). Participants with renal failure (estimated glomerular filtration rate < 30 mL/min) or any gastrointestinal, endocrine, haematological, cardiovascular, respiratory, psychiatric, or central nervous system diseases, active cancer within the last five years, or acute inflammation or infection were excluded, as were pregnant or lactating women. Participants were recruited through advertisements at Monash Medical Centre, Monash University, and within the community via the Australian National Diabetes Service Scheme. The study included one screening visit and two clinic visits (one before and one after the intervention).

### 2.2. Ethics

This RCT was carried out at a single site (Monash Health Translational Research Facility, Melbourne, Australia) and was conducted according to the principles outlined in the Declaration of Helsinki [24]. A protocol was published to ensure transparency [25], with the trial methodology aligned with the Standardised Protocol Interventions: Recommendations for Interventional Trials (SPIRIT) 2013 Statement [26] and reported according to CONSORT guidelines [27]. The trial received approval from both the Monash Health Human Research Ethics Committee (Ref. No. 16061A) and Monash University (ID No. 7787), Melbourne, Australia and was registered on clinicaltrials.gov (NCT02917928, 28/09/16). All participants provided written informed consent.

### 2.3. Screening and Baseline Assessments

At visit 1, after obtaining informed consent, participants underwent a screening visit with a medical exam including a demographic questionnaire, anthropometric measures, and medical history, administered by the trial’s medical practitioner. A urine pregnancy test was performed to exclude any unknown pregnancies in women. Next, participants underwent a 2 h oral glucose tolerance test (OGTT) after a 10–12 h overnight fast to assess glucose tolerance and classify participants as having either normal glucose tolerance, prediabetes, or type 2 diabetes [28]. Fasting blood samples were collected via venepuncture. After centrifugation, the samples were frozen at −80 °C for later analyses by Monash Health Pathology. The second visit involved baseline body composition, cardiovascular, and cognitive assessments, followed by completing validated questionnaires to assess physical activity and dietary habits (detailed below).

### 2.4. Randomisation and Blinding

A random assignment was conducted using a computerised random-sequence generation program and organised in blocks of four by gender and metformin use to ensure balanced group allocation. Randomisation codes, created by the study statistician who was blinded to the allocation, were sent to the clinical trial pharmacist who dispensed the medication. All researchers, nurses, and other staff members involved in the trial, including laboratory technicians, were blinded to participant allocation. The clinical trials pharmacist only disclosed the codes after trial completion and data analysis of the primary outcomes.

### 2.5. Intervention and Monitoring

Participants who met eligibility criteria were randomly assigned to either the carnosine group, receiving two capsules of 500 mg carnosine twice daily (CarnoPure, Flamma S.p.A, Bergamo, Italy), or the placebo group (methylcellulose), receiving an equivalent number of identical placebo capsules for 14 weeks. This treatment dose and duration were based on the previous pilot trial investigating the effect of carnosine supplementation on glucose metabolism [22]. The purity of carnosine was laboratory tested to ensure greater than 99.5% freedom from contaminants. Supplements were distributed in identical capsules and containers to ensure both participants and researchers were blinded to group allocation. Participants were instructed to maintain their usual diet and physical activity levels during the trial period and to return any unused capsules at the 14-week appointment to ascertain adherence to the dosing protocol.

Monthly phone calls were made, where participants were assessed for adverse events associated with supplementation. Participants were also asked to contact our researchers to report fatigue, dry mouth, and rash if these symptoms developed between the visits and phone calls. To assess medication adherence, participants were asked to return the supplement containers at the end of the study.

### 2.6. Outcome Measures

Multiplex assays (10-plex obesity panel) were used to measured adiponectin, adipsin, resistin, serpin E1/plasminogen activator inhibitor-1 (PAI-1), leptin, CRP, IL-6, IL-10, monocyte chemoattractant protein-1 (MCP-1), and TNF-α, as per the manufacturer’s protocol (catalog # LOBM000, R&D systems, Minneapolis, MN, USA). Briefly, all reagents were brought to room temperature (RT) prior to use. A standard cocktail and samples were prepared using Calibrator Diluent RD6-46. Following the protocol, magnetic beads, biotin-antibody, and streptavidin-PE were prepared. Fifty microlitres of standards or samples were pipetted in duplicate, followed by 50 µL of diluted micro particle cocktail in each well. The plate was incubated for 3h at RT in a microplate shaker with a speed of 800 ± 50 rpm. Plates were washed three times using the magnetic washer and incubated with 50 µL biotin-antibody cocktail for 1h at RT in a shaker. The washing step was repeated three times, and 50 µL of diluted streptavidin-PE was added to each well and incubated for 30 min at RT. After incubation, plates were washed, and the microparticle complex was suspended in 100 µL of wash buffer. The plates were read using a Bioplex 200 array reader (Bio-Rad Laboratories, Hercules, CA, USA) [25]. Laboratory personnel who performed the Bioplex assay were blinded to intervention/placebo status. Hence, samples were randomly ordered and were run in the same batch (96-well plate). All inter- and intra-assay coefficients of variation (CVs) varied from 0% to 15% for all biomarkers, indicating a good laboratory performance.

For anthropometric assessments, a digital scale (Tanita BWB-600, Tanita, Tokyo, Japan) and wall-mounted stadiometer (Seca 206, Seca, Hamburg, Germany) were used to measure body weight (kg) and height (cm), respectively. BMI was determined using the formula weight (kg)/height squared (m^2^). Waist circumference was also measured at the midpoint between the upper iliac crest and the lowermost rib at the end of a normal expiration using a constant-tension tape. Body composition parameters, including total body fat percentage and visceral adipose tissue, were estimated using whole-body dual-energy X-ray absorptiometry (DXA) (Hologic Discovery A, Hologic, Marlborough, MA, USA). The DXA scanner was calibrated daily with the manufacturer’s spine phantom and the CV for fat mass and visceral adipose tissue were <0.96% and 4.7%, respectively.

Participants filled out questionnaires about general demographic information, physical activity levels, and dietary intake. The physical activity was measured using a short form of the international physical activity questionnaire (IPAQ) and dietary intake was assessed through food records of three consecutive days. Food records were analysed using Foodworks.online.V.2.0 Professional Dietary Software (Brisbane, Australia, Xyris Pty Ltd.), along with Australian food composition data (NUTTAB 2010).

### 2.7. Statistical Analyses

The sample size calculation was based on the primary outcomes of blood glucose level and HbA1c as reported in the published primary trial results [29,30]. The primary analysis followed per-protocol principles, while additional intention-to-treat (ITT) analyses were conducted as sensitivity analyses (Supplementary File) using multiple imputation of missing data (SPSS Inc., Chicago, IL, USA, version 24). Shapiro–Wilk tests, scatterplots, and histograms were used to assess normality, and continuous variables were logarithmically transformed to base 10 to approximate a normal distribution. Parametric data were reported as mean ± standard deviation (SD) and non-parametric data were reported as median and interquartile range (IQR). Baseline differences in characteristics between groups were assessed by independent sample t-tests and chi-square tests as appropriate. Changes in outcome variables (follow-up—baseline) were calculated (delta) and differences in both delta and follow-up values between groups were assessed by independent t-tests. Multivariable linear regression was performed to determine if the differences between the two groups were independent of clinically relevant variables, as predetermined in our published protocol [25]. Further exploratory analyses were conducted in pre-specified subgroups based on diabetic status and metformin intake. All tests were two-tailed, and the alpha level of significance was set at *p* < 0.05.

## 3. Results

**Figure 1:**
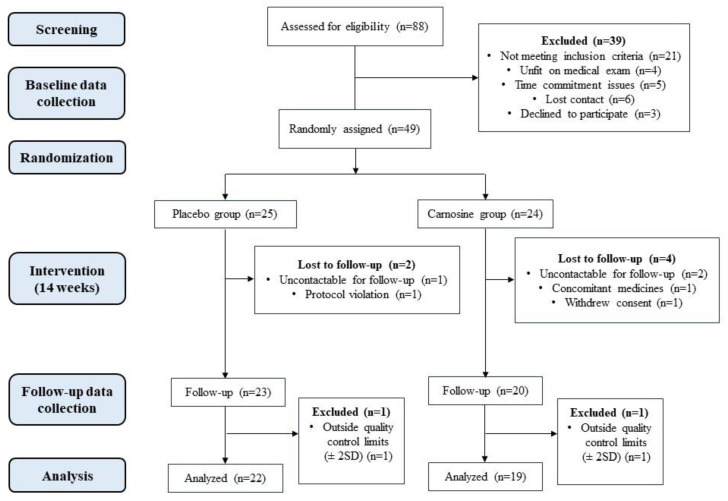
Study design and flow of participants.

Of the 88 participants who attended medical reviews, 49 participants were randomly assigned to either the placebo group (*n* = 25) or carnosine group (*n* = 24). Six participants (*n* = 2 from the placebo group and n = 4 from the carnosine group) were excluded or withdrew due to being uncontactable for follow-up (*n* = 3), using concomitant medications (*n* = 1), protocol violation (*n* = 1), or withdrawing consent (*n* = 1). The laboratory results for two additional participants were beyond two standard deviations for several inflammatory markers, indicating possible active infection. Thus, they were excluded from this analysis. In total, 41 participants (*n* = 22 in the placebo group and *n* = 19 in the carnosine group) were analysed in the present study in a per-protocol fashion (Figure 1). There were no adverse effects reported in the study.

### 3.1. Sample Characteristics

The baseline characteristics of the study participants are outlined in Table 1 (Table S1 using ITT appproach). The sample comprised 29 males and 12 females, aged 53 (42.6, 59.3) years [median (IQR)], with a mean BMI of 29.4 ± 4.03 kg/m^2^. Based on OGTTs, 22 participants had prediabetes and 19 had T2D. At baseline, there were no significant differences between the two treatment groups in demographic characteristics including age, sex, ethnicity, and family history of diabetes.

### 3.2. Differences in Outcomes Between Treatment Groups

Table 2 shows baseline, follow-up, and change (delta) values for all outcome measures in the placebo and carnosine groups. There were no significant differences in changes in MCP-1, CRP, IL-6, IL-10, and TNF-α levels between the carnosine and placebo groups. Similarly, carnosine supplementation did not change any of the adipokine levels, including adiponectin, adipsin, leptin, resistin, and serpin, compared with placebo. Similar results were obtained using ITT analyses, which are presented in Table S2.

In the multivariable regression analyses, differences remained non-significant after adjustment for covariates (Table 3). In model 1, after adjustment for age and sex, there were no differences in changes in outcome measures between carnosine and placebo groups. Adding anthropometric measures to the model, including body fat percentage (Model 2) or visceral adipose tissue (Model 3), did not alter the results, nor did replacing these measures with BMI or waist circumference. The results from the ITT analyses were consistent with these findings (Table S3).

Exploratory subgroup analyses were conducted in individuals with prediabetes and those with T2D, as well as in individuals who were using or not using metformin. Findings remained non-significant for all pro- or anti-inflammatory markers and adipokines measured in each subgroup, using either per-protocol (Table 4) or ITT analyses (Table S4).

## 4. Discussion

This is the first analysis of a randomised, placebo-controlled, double-blind trial examining the effects of 2 g/day of oral carnosine supplementation for 14 weeks on inflammatory biomarkers in individuals with prediabetes and T2D. The results showed no differences in the inflammation markers and adipokines measured, including MCP-1, IL-6, IL-10, TNF-α, CRP, adiponectin, leptin, adipsin, resistin, and serpin after carnosine supplementation compared with placebo. The results were unchanged in multivariable analyses adjusted for age, sex, and anthropometric measures, or in exploratory subgroup analyses by prediabetes/diabetes status and by metformin use.

Our results for CRP and TNF-α are inconsistent with a recent systematic review and meta-analysis by our group, in which carnosine/HCDs supplementation reduced CRP and TNF-α levels, but not adiponectin or IL-6. However, the meta-analysis included nine clinical trials of mixed population groups (children and adults; healthy and with existing diseases; *n* = 350), and study heterogeneity and low certainty of evidence were important limitations, with pooled analyses using a variety of HCDs (e.g., anserine, histidine). These factors may explain the discrepancies between the meta-analysis and the present study results. Further, only two of the nine RCTs identified in the meta-analysis examined the effects of carnosine alone on inflammatory markers in adults [21,23], with only one in T2D, underscoring the need for additional clinical trials in this context. The single trial in individuals with T2D (*n* = 44) by Houjeghani et al. [23] measured three inflammation markers, and found that carnosine significantly reduced serum concentrations of TNF-α, but not IL-1β or IL-6, following 1 g/day carnosine (capsule) for 12 weeks (compared with 2 g/day for 14 weeks in the present study). Unlike the present study, there was no exclusion by HbA1c levels in the previous study [23]. Hence, it is possible that some participants in their study had higher HbA1c and/or poorly controlled diabetes with poorer baseline inflammation, and were therefore more likely to benefit more from carnosine supplementation [23]. It is also possible that diabetes medications used in the study by Houjeghani et al. [23] may have contributed to the positive effects on TNF-α, whereas our study excluded medications other than metformin and explored metformin use in the subgroup analysis. Moreover, Houjeghani et al. [23] only included participants from a single ethnic group (Iran), whereas we included a multiracial participant group. The limited number and heterogenous nature of trials examining the effects of carnosine on these markers precludes definitive conclusions and highlights the need for further research to address these inconsistencies in the evidence.

We found no effect of carnosine supplementation on any of the other markers measured, including MCP-1, IL-10, adiponectin, adipsin, leptin, resistin, and serpin. To the best of our knowledge, this is the first study to investigate the effects of carnosine supplementation on these markers in individuals with prediabetes and T2D. A previous pilot trial by our group in a non-diabetic overweight/obese population (n = 24) used the same dose of carnosine supplementation (2 g/day, 12 weeks) and showed improved serum resistin, but no effects on leptin, adiponectin, or adipsin levels [21], consistent with the present data. It is possible that carnosine preferentially affects certain types of adipose tissue or specific cells within adipose tissue (e.g., macrophages that secrete resistin); however, this hypothesis is not supported by the present data and further study is required. Findings from experimental studies have also been conflicting; 1000 mg/kg of carnosine supplementation for 169 days did not reduce circulating levels of MCP-1 in healthy cats [31], while the mRNA expression of the genes for MCP-1 were significantly attenuated by a 30 mg/kg FL-926-16 (a novel carnosinase-resistant derivative of carnosine) treatment for 14 weeks in diabetic mice [32]. A mixed supplement (carnosine with α-lipoic acid) [33] decreased MCP-1 levels in the brains of obese rats; however, it is difficult to isolate the effect of carnosine or extrapolate these in vitro animal data to the in vivo human context. When comparing our study with previous studies, we note that there are key differences in study populations (e.g., ethnicity and/or health status), interventions (e.g., form, dose, and duration), and biomarkers measured. Overall, it is evident that while our study adds valuable data to the existing literature on carnosine supplementation in individuals with prediabetes and T2D, the findings remain inconclusive regarding its impact on inflammatory markers and adipokines. This is largely due to differences in study populations, intervention regimens/dosages, baseline risk profiles, baseline levels of biomarkers such as MCP-1, resistin, and TNF-α, which varied between groups, and concomitant medications, emphasising the need for future studies to focus on larger, well-characterised cohorts with stratification by disease status and baseline inflammation levels.

To our knowledge, despite these variations, this is the first RCT to investigate the effects of carnosine supplementation, without co-interventions, on a range of pro- and anti-inflammatory mediators in individuals with prediabetes and T2D. The trial incorporated a rigorous methodology in line with international guidelines, including a double-blinded randomised placebo-controlled design. We provided a therapeutic dose of carnosine based on our pilot data, which is higher than most previous studies, for a duration deemed sufficient to assess its potential effects on inflammatory markers. However, some limitations should be noted. First, this is a secondary analysis of a previous RCT [29], where the sample size calculation was based on changes in blood glucose concentrations and not on changes in inflammatory markers. Therefore, the sample size of this exploratory study may have been insufficient to detect differences in these markers. Second, we administered 2 g/day based on previous studies, as described in the published study reporting the primary outcomes of this trial [29]. However, we acknowledge that using only one dose limits our ability to detect a dose–response relationship. Third, although high-molecular weight adiponectin is considered the metabolically active form, we measured total adiponectin in this study [34]. Fourth, we did not measure urinary carnosine, a measure which would be helpful to assess the absorption of the supplemented carnosine. Finally, the compliance evaluation relied on returned bottles, with some participants failing to return them.

## 5. Conclusions

Our findings suggest that carnosine supplementation has no effect on circulating pro- and anti-inflammatory cytokines and adipokines in participants with prediabetes or well-controlled T2D. These results are incongruent with the anti-inflammatory properties of carnosine reported in in vitro and animal models, suggesting that carnosine may function differently in the human in vivo context; however, further confirmatory data are needed to corroborate these findings. Future studies should focus on different intervention doses and larger, well-characterised cohorts with a range of baseline risk profiles and inflammation levels. This will enable appropriate stratification and sufficient statistical power to address the current evidence gaps and determine the therapeutic potential of carnosine for mitigating inflammation.

## Figures and Tables

**Table 1:** Participant demographics and baseline characteristics.

Characteristic	Placebo Group (*n* = 22)	Carnosine Group (*n* = 19)
Age, years	50.2 (42.1, 59.3) ^a^	54.5 (45.4, 59.5)
Female, *n* [%]	6 (27.3)	6 (31.6)
Caucasian	11 (50)	8 (42.1)
South and Central Asian	4 (18.2)	5 (26.3)
Southeast and Northeast Asian	6 (27.3)	4 (21)
Other ^b^	1 (4.5)	2 (10.5)
Prediabetic, *n* [%]	11 (50)	11 (57.9)
Diabetic, *n* [%]	11 (50)	8 (42.1)
Obese (BMI > 30 [kg/m^2^]), *n* [%]	5 (22.7)	9 (47.4)
Family history of diabetes ^c^, *n* [%]	4 (18.2)	5 (26.3)
Treated with metformin, *n* [%]	9 (40.9)	7 (36.8)
Total energy ^d^ [KJ]	8095.5 ± 1315.5	8439.5 ± 1974.5
Physical activity, IPAQ-METS score ^e^	1359 (426, 3508.3)	1816 (817.5, 4878)
Weight, [kg]	83.5 ± 12.2	86.7 ± 23.9
Height, [cm]	169.8 ± 9.8	170.1 ± 10.5
BMI, [kg/m^2^]	28.9 ± 3.1	29.9 ± 4.9
WC, [cm]	99 ± 8.8	102.2 ± 14.8
Percentage of body fat [%]	36.6 ± 7.2	35.8 ± 7.7
Visceral adipose tissue [kg]	154.6 ± 44.9	140.8 ± 34.8
HbA1c, [%]	6.7 ± 0.8	6.5 ± 0.6

^a^ All such median (IQR) values are presented for non-normally distributed variables. Variables with non-normal distributions were log transformed to base 10 prior to analysis. ^b^ Refers to Middle Eastern, Polynesian, African, and South American ethnicities. ^c^ Includes only first-degree relative with diabetes. ^d^ Calculated from food records and self-reported questionnaires. ^e^ IPAQ-METS, international physical activity questionnaire—metabolic equivalent (multiples of the resting metabolic rate). Abbreviations: BMI: body mass index, WC: waist circumference, HbA1c: haemoglobin A1c.

**Table 2:** Comparison of inflammatory markers and adipokines before and after supplementation in both groups.

Outcome Variable	Placebo Group (*n* = 22)			Carnosine Group (*n* = 19)			*p*_1_	*p*_2_
	Baseline	Follow-Up	Change	Baseline	Follow-Up	Change		
Adiponectin, [μg/mL]	9.1 ± 3.3	8.2 ± 4.2	−0.9 ± 2.9	9.5 ± 4.9	8.1 ± 5.2	−1.4 ± 3.6	0.95	0.68
MCP-1, [pg/mL]	279.4 ± 85.4	255.6 ± 90.2	−23.8 ± 61.1	296.9 ± 101.1	269.5 ± 95	−27.5 ± 92.5	0.63	0.88
CRP, [ng/mL]	8.5 (3.3, 26.2) ^a^	5.6 (3.7, 19.2)	−0.4 (−6.9, 10.5)	12.6 (4.2, 19.5)	10.2 (3.7, 17.3)	0.5 (−9.1, 7.7)	0.54	0.55
Complement Factor D/Adipsin, [μg/mL]	4 ± 1.5	3.9 ± 1.8	−0.04 ± 1.3	4.1 ± 1.6	4 ± 1.8	−0.01 ± 1.4	0.91	0.94
Leptin, [ng/mL]	21.9 (10.7, 33.8)	18.5 (8.6, 32.7)	−0.5 (−3.9, 2.6)	13 (9.6, 41)	15.4 (7.7, 28.7)	−2.4 (−8.9, 0.8)	0.97	0.12
Resistin, [ng/mL]	6.2 ± 3.3	5.4 ± 2.5	−0.8 ± 2	4.8 ± 1.7	4.4 ± 2	−0.5 ± 1.8	0.14	0.59
Serpin E1/PAI-1, [ng/mL]	111.6 ± 41.4	90.9 ± 35.3	−20.6 ± 37.1	104 ± 45.9	89.4 ± 39.1	−14.6 ± 42.8	0.89	0.63
IL-6, [pg/mL]	2.5 ± 0.7	2.5 ± 0.6	0.02 ± 0.6	2.8 ± 0.9	2.7 ± 0.8	−0.1 ± 1.2	0.46	0.66
IL-10, [pg/mL]	1.1 ± 0.2	1 ± 0.3	−0.1 ± 0.3	1.1 ± 0.2	1 ± 0.3	−0.04 ± 0.3	0.86	0.78
TNF-α, [pg/mL]	5.1 ± 2.3	4.9 ± 1.7	−0.2 ± 1.1	6.1 ± 2.4	6.1 ± 2.2	0.04 ± 1.5	0.06	0.61

Data presented as mean ± standard deviation unless otherwise specified. ^a^ All such median (IQR) values are presented for non-normally distributed variables. Variables with non-normal distributions were log transformed to base 10 prior to analysis. *p* values of independent t-tests for differences at follow-up (*p*_1_) or in change scores (*p*_2_) between groups. Abbreviations: MCP-1: monocyte chemoattractant protein-1, CRP: c-reactive protein, PAI-1: plasminogen activator inhibitor-1, IL-6: interleukin-6, IL-10: interleukin-10, TNF-α: tumour necrosis factor-α.

**Table 3:** Multivariable regression analysis for differences in metabolic variables between carnosine and placebo groups after adjustment for covariates ^1^.

Dependent Variable ^2^	Models	*β*	95% CI	SE	*R* ^2^	*p*
Change in adiponectin, [μg/mL]	Model 1	−0.5	−2.6, 1.6	1.04	0.04	0.65
	Model 2	−0.3	−2.5, 1.8	1.05	0.07	0.74
	Model 3	−0.3	−2.6, 2	1.1	0.02	0.78
Change in MCP-1, [pg/mL]	Model 1	−3.9	−45, 37.2	20.3	0.1	0.84
	Model 2	−4.8	−46.7, 37.1	20.7	0.1	0.82
	Model 3	−17.9	−71.7, 35.7	26.4	0.1	0.50
Change in CRP, [ng/mL]	Model 1	−1.1	−15.1, 12.9	6.9	0.005	0.88
	Model 2	−2.5	−16.2, 11.2	6.8	0.1	0.71
	Model 3	6.5	−12.4, 25.5	9.3	0.1	0.48
Change in complement factor D/adipsin, [μg/mL]	Model 1	−0.2	−0.9, 0.5	0.4	0.1	0.52
	Model 2	−0.2	−0.9, 0.5	0.4	0.1	0.54
	Model 3	−0.04	−1.01, 0.9	0.5	0.1	0.93
Change in leptin, [ng/mL]	Model 1	−7.6	−16.8, 1.6	4.5	0.1	0.10
	Model 2	−8.4	−17.5, 0.7	4.5	0.2	0.07
	Model 3	−11.3	−24.1, 1.6	6.3	0.2	0.08
Change in resistin, [ng/mL]	Model 1	0.4	−0.7, 1.5	0.5	0.04	0.49
	Model 2	0.3	−0.8, 1.5	0.5	0.05	0.54
	Model 3	0.2	−1.2, 1.6	0.7	0.03	0.77
Change in serpin E1/PAI-1, [ng/mL]	Model 1	3.04	−21.5, 27.6	12.1	0.1	0.80
	Model 2	4.6	−20.1, 29.2	12.2	0.1	0.70
	Model 3	3.6	−23.5, 30.6	13.3	0.1	0.79
Change in IL-6, [pg/mL]	Model 1	−0.1	−0.7, 0.4	0.3	0.02	0.60
	Model 2	−0.1	−0.8, 0.4	0.3	0.02	0.56
	Model 3	−0.3	−0.9, 0.3	0.3	0.1	0.35
Change in IL-10, [pg/mL]	Model 1	0.01	−0.1, 0.2	0.1	0.02	0.87
	Model 2	0.02	−0.1, 0.2	0.1	0.04	0.79
	Model 3	0.1	−0.1, 0.3	0.1	0.1	0.38
Change in TNF-α, [pg/mL]	Model 1	0.2	−0.6, 1.1	0.4	0.01	0.59
	Model 2	0.2	−0.6, 1.1	0.4	0.02	0.55
	Model 3	0.04	−0.8, 0.9	0.4	0.07	0.92

^1^ Model 1 was adjusted for age and sex. Model 2 was adjusted for age, sex, and percentage of body fat. Model 3 was adjusted for age, sex, and visceral adipose tissue. *p* values were determined with the use of a multiple linear regression analysis (ANCOVA) for differences between groups after adjustment for covariates. ^2^ Data presented as unstandardised beta-coefficients (*β*), confidence interval (CI), standard error (SE), and adjusted R-square (*R*^2^) values with corresponding *p* values for differences in change values in adipokine concentrations between groups, after adjustment for covariates. Abbreviations: MCP-1: monocyte chemoattractant protein-1, CRP: c-reactive protein, PAI-1: plasminogen activator inhibitor-1, TNF-α: tumour necrosis factor-α, IL-6: interleukin-6, IL-10: interleukin-103.3. Exploratory subgroup analysis.

**Table 4:** Subgroup analyses of participants with prediabetes/diabetes and participants taking metformin intake vs. on diet only.

Outcome Variable	Metformin (+) (*n* = 16)			Metformin (−) (*n* = 25)			Prediabetes (*n* = 22)			Diabetes (*n* = 19)		
	Placebo Group (*n* = 9)	Carnosine Group (*n* = 7)	*p*	Placebo Group (*n* = 13)	Carnosine Group (*n* = 12)	*p*	Placebo Group (*n* = 11)	Carnosine Group (*n* = 11)	*p*	Placebo Group (*n* = 11)	Carnosine Group (*n* = 8)	*p*
Change in adiponectin, [μg/mL]	−0.6 ± 3.7 ^a^	−2.2 ± 3.6	0.41	−1.2 ± 2.4	−0.8 ± 3.7	0.81	−0.6 ± 3.6	−2.2 ± 3.3	0.31	−1.2 ± 2.2	−0.2 ± 3.9	0.48
Change in MCP-1, [pg/mL]	−9.3 ± 49.5	−88 ± 97.8	0.08	−33.8 ± 68.02	7.8 ± 71.5	0.14	−0.3 ± 50.2	−20.3 ± 86.4	0.51	−47.4 ± 63.9	−37.3 ± 105.7	0.79
Change in CRP, [ng/mL]	−2.2 (−8.7, 8.7) ^b^	1.1 (−9.3, 3.7)	0.86	−0.03 (−7.1, 11.4)	0.5 (−4.4, 17.9)	0.54	−2.2 (−11.5, −0.03)	0.4 (−5, 31.7)	0.25	1.3 (−2.8, 13.7)	1.3 (−19.7, 6.7)	0.35
Change in complement Factor D/adipsin, [μg/mL]	0.3 ± 1.2	0.2 ± 2.2	0.92	−0.3 ± 1.3	−0.1 ± 0.8	0.77	0.1 ± 1.3	−0.5 ± 0.8	0.16	−0.2 ± 1.3	0.7 ± 1.8	0.21
Change in leptin, [ng/mL]	0.05 (−3.6, 2.03)	−6.1 (−12.4, −2.2)	0.10	−1.4 (−6.9, 2.9)	0.5 (−3.9, 3.1)	0.67	−1.1 (−4.2, 7.1)	−2.2 (−3.9, 0.8)	0.31	0.05 (−3.7, 1.3)	−4.8 (−11.5, 3)	0.19
Change in resistin, [ng/mL]	−1.3 ± 3.03	−0.4 ± 2.5	0.53	−0.4 ± 0.8	−0.5 ± 1.3	0.87	−0.6 ± 2.4	−0.1 ± 1.7	0.55	−0.9 ± 1.6	−0.9 ± 1.9	0.98
Change in serpin E1/PAI-1, [ng/mL]	−16.6 ± 44.7	−22.2 ± 50.3	0.81	−23.4 ± 32.4	−10.2 ± 39.4	0.37	−25.5 ± 34.2	−0.1 ± 1.7	0.88	−15.7 ± 40.7	−2.7 ± 47.7	0.53
Change in IL-6, [pg/mL]	−0.1 ± 0.6	−0.2 ± 1.9	0.88	0.1 ± 0.5	−0.1 ± 0.6	0.48	−0.08 ± 0.5	−0.5 ± 0.8	0.17	0.1 ± 0.6	0.5 ± 1.4	0.50
Change in IL-10, [pg/mL]	−0.1 ± 0.4	0.02 ± 0.2	0.45	−0.05 ± 0.3	−0.1 ± 0.3	0.82	−0.1 ± 0.3	−0.1 ± 0.3	0.84	0.03 ± 0.3	0.1 ± 0.1	0.59
Change in TNF-α, [pg/mL]	−0.1 ± 1.1	0.1 ± 1.3	0.70	−0.2 ± 1.1	0.0 ± 1.7	0.72	0.2 ± 0.8	−0.3 ± 1.1	0.25	−0.5 ± 1.2	0.5 ± 1.9	0.16

^a^ Data are expressed as mean ± SD for change scores. All analyses performed using independent *t*-test for differences between groups. ^b^ All such median (IQR) values are presented for non-normally distributed variables. Variables with non-normal distributions were log transformed to the base 10 prior to analysis. Abbreviations: CRP: c-reactive protein, PAI-1: plasminogen activator inhibitor-1, MCP-1: monocyte chemoattractant protein-1, TNF-α: tumour necrosis factor-α, IL-6: interleukin-6, IL-10: interleukin-10.

## Data Availability

The datasets utilised and analysed in this study can be requested from the corresponding author upon reasonable request.

## Supplementary Materials

The following supporting information can be downloaded at: https://www.mdpi.com/article/10.3390/nu16223900/s1, Table S1: Participant demographics and baseline characteristics; Table S2: Comparison of inflammatory markers and adipokines before and after supplementation in both groups; Table S3: Multivariable regression analysis for differences in metabolic variables between carnosine and placebo groups after adjustment for covariates; Table S4: Subgroup analyses of participants with prediabetes/diabetes and participants taking metformin intake vs. on diet only.
