# Supplementary material for: Carnosine Supplementation Has No Effect on Inflammatory Markers in Adults with Prediabetes and Type 2 Diabetes: A Randomised Controlled Trial
Source: Nutrients. 2024 Nov 15;16(22):3900. doi: 10.3390/nu16223900 (PMC11597812; doi:10.3390/nu16223900)
Supplement: Supplementary file 1 [file nutrients-16-03900-s001.zip › nutrients-3313682-supplementary.pdf]

Table S1. Participant demographics and baseline characteristics

Characteristic	Placebo group (n= 24)	Carnosine group (n= 24)
Age, years	50.2 (42.4, 59.1) [a]	52 (46.1, 59.5)
Female, *n* [%]	6 (25)	10 (41.6)
Caucasian	11 (45.8)	15 (62.5)
South and Central Asian	8 (33.3)	6 (25)
Southeast and Northeast Asian	4 (16.6)	3 (12.5)
Other[b]	1 (4.1)	-
Prediabetic, *n* [%]	12 (50)	16 (66.6)
Diabetic, *n* [%]	12 (50)	8 (33.3)
Obese (BMI>30 [kg/m^2]), *n* [%]	5 (20.8)	10 (41.6)
Family history of diabetes[e], *n* [%]	4 (16.6)	5 (20.8)
Treated with metformin, *n* [%]	10 (41.6)	8 (33.3)
Total energy, kj	8074.1±1260.6	8426.9±1793.2
Physical activity[c], IPAQ-METS score[d]	1519.5 (549.7, 3775.8)	2932.3 (1386, 4831.5)
Weight, [kg]	82.9±15.1	89.5±22.2
Height, [cm]	169.4±10.2	172.4±10.9
BMI, [kg/m^2]	28.9±2.9	29.7±4.3
WC, [cm]	98.4±10.4	103.3±13.1
Percentage of body fat [%]	36.8±6.9	35.9±6.9
Visceral adipose tissue [kg]	153.7±46.1	136±36.2
HbA1c, [%]	6.7±0.8	6.5±0.6

[a] Median; IQR in in parentheses (all such values for nonnormally distributed variables). Nonnormally distributed variables were log transformed to the base 10 before analysis.
[b] Refers to African, Middle Eastern, South American, and Polynesian ethnicities.
[c] Calculated from self-reported questionnaires and food records.
[d] IPAQ-METS, international physical activity questionnaire–multiples of the resting metabolic rate.
[e] Includes only first-degree relative with diabetes.
[*]Abbreviations: BMI: body mass index, IPAQ-METS: international physical activity questionnaire-metabolic equivalent, WC: waist circumference, HbA1c: haemoglobin A1c.

Table S2. Comparison of inflammatory markers and adipokines before and after supplementation in both groups

Outcome variable	Placebo group (n= 24)			Carnosine group (n= 24)			P_1	P_2
	Baseline	Follow-up	Change	Baseline	Follow-up	Change		
Adiponectin, [µg/ml]	8.7±2.7	7.7±3.2	-0.9±2.8	9.2±4.1	7.8±4.2	-1.4±3.2	0.95	0.64
MCP-1, [pg/ml]	280.4±81.7	256.3±86.2	-24.1±58.4	292.9±77	268.9±84.1	-24±62.5	0.60	0.99
CRP, [ng/ml]	9.1 (3.4, 27.4) [a]	8.01 (4.1, 23.1)	-0.2 (-4.8, 8.05)	15.3 (4.4, 27.4)	14.2 (5.7, 30.7)	1.6 (-4.4, 3.6)	0.45	0.88
Complement Factor D/Adipsin, [µg/ml]	4±1.4	4±1.7	0.03±1.1	4.1±1.4	4.05±1.6	-0.1±1.1	0.97	0.67
Leptin, [ng/ml]	23.6 (11.7, 31.4)	20.6 (9.3, 30.7)	-1.2 (-3.8, 2.3)	25.7 (10.2, 30.9)	26.9 (10.1, 27.8)	-3.1 (-5.6, -3.1)	0.84	0.14
Resistin, [ng/ml]	6.01±3.01	5.4±2.3	-0.7±1.8	4.9±1.5	4.6±1.8	-0.3±1.3	0.20	0.45
Serpin E1/PAI-1, [ng/ml]	109.3±35.5	88.8±33.1	-20.5±35.4	105.04±40.6	89.5±34.6	-15.5±37.9	0.93	0.64
IL-6, [pg/ml]	2.6±0.7	2.5±0.5	-0.01±0.6	2.8±0.7	2.7±0.8	-0.1±1.1	0.44	0.79
IL-10, [pg/ml]	1.1±0.2	1.1±0.2	-0.04±0.3	1.1±0.2	1.1±0.2	-0.002±0.2	0.86	0.54
TNF-α, [pg/ml]	5.1±2.2	4.9±1.6	-0.2±1.05	5.8±1.6	5.8±1.8	0.006±1.4	0.06	0.62

Data presented as mean ± standard deviation unless otherwise specified. Non-normally distributed variables were log-transformed to the base 10 to approximate normality prior to analyses.

[a] Median; IQR in in parentheses (all such values for nonnormally distributed variables). Nonnormally distributed variables were log transformed to the base 10 before analysis.
P values independent *t* tests for differences at follow-up (P_1) or in change scores (P_2) between groups

[*]Abbreviations: CCL2: chemokine (C-C motif) ligand 2, MCP-1: monocyte chemoattractant protein-1, CRP: c-reactive protein, PAI-1: plasminogen activator inhibitor-1, IL-6: interleukins-6, IL-10: interleukins-10, TNF-α: tumour necrosis factor-α

Table S3. Multivariable regression analysis for differences in metabolic variables between carnosine and placebo groups after adjustment for covariates[1]

Dependent Variable[2]	Models	*β*	95% CI	SE	R^2	*P*
Change in adiponectin, [μg/ml]	Model 1	-0.3	-2.1, 1.4	0.9	0.04	0.69
	Model 2	-0.2	-2.1, 1.6	0.9	0.05	0.82
	Model 3	-0.4	-2.3, 1.5	0.9	0.04	0.67
Change in MCP-1, [pg/ml]	Model 1	-6.4	-41.7, 28.9	17.4	0.07	0.71
	Model 2	-6.8	-43.2, 29.6	18.1	0.07	0.70
	Model 3	-12.2	-47.8, 23.3	17.6	0.1	0.49
Change in CRP, [ng/ml]	Model 1	-0.6	-12.6, 11.4	5.9	0.003	0.91
	Model 2	-2.7	-14.6, 9.2	5.9	0.07	0.64
	Model 3	0.1	-12.3, 12.4	6.1	0.01	0.99
Change in complement factor D/adipsin, [μg/ml]	Model 1	-0.2	-0.8, 0.4	0.3	0.07	0.52
	Model 2	-0.2	-0.8, 0.4	0.3	0.07	0.54
	Model 3	-0.2	-0.8, 0.4	0.3	0.07	0.52
Change in leptin, [ng/ml]	Model 1	-6.8	-14.7, 1.1	3.9	0.1	0.09
	Model 2	-8.1	-15.9, -0.1	3.9	0.1	0.05
	Model 3	-7.5	-15.6, 0.6	4.1	0.1	0.07
Change in resistin, [ng/ml]	Model 1	0.3	-0.6, 1.3	0.5	0.03	0.50
	Model 2	0.3	-0.7, 1.2	0.5	0.04	0.57
	Model 3	0.4	-0.6,1.3	0.5	0.03	0.45
Change in serpin E1/PAI-1, [ng/ml]	Model 1	2.6	-18.5, 23.7	10.5	0.1	0.80
	Model 2	4.9	-16.5, 26.4	10.6	0.1	0.64
	Model 3	2.5	-19.3, 24.4	10.8	0.1	0.81
Change in IL-6, [pg/ml]	Model 1	-0.1	-0.6, 0.4	0.3	0.02	0.65
	Model 2	-0.1	-0.7, 0.4	0.3	0.04	0.53
	Model 3	-0.1	-0.7, 0.3	0.3	0.06	0.47
Change in IL-10, [pg/ml]	Model 1	0.04	-0.1, 0.2	0.07	0.01	0.62
	Model 2	0.04	-0.1, 0.2	0.07	0.02	0.55
	Model 3	0.03	-0.1, 0.2	0.07	0.02	0.69
Change in TNF-α, [pg/ml]	Model 1	0.2	-0.5, 0.9	0.4	0.02	0.56
	Model 2	0.3	-0.5, 1.05	0.4	0.04	0.45
	Model 3	0.1	-0.6, 0.9	0.4	0.03	0.68

[1] Model 1 was adjusted for age and sex. Model 2 was adjusted for age, sex, and percentage of body fat. Model 3 was adjusted for age, sex, visceral adipose tissue. P values were determined with the use of a multiple linear regression analysis (ANCOVA) for differences between groups after adjustment for covariates.

[2] Data presented as unstandardized beta-coefficients (β), confidence interval (CI), standard error (SE), and adjusted R-square (R^2) values with corresponding p values for differences in change values in adipokine concentrations between groups, after adjustment for covariates.

Abbreviations: MCP-1: monocyte chemoattractant protein-1, CRP: c-reactive protein, PAI-1: plasminogen activator inhibitor-1, IL-6*:* interleukins-6, IL-10: interleukins-10, TNF-α: tumour necrosis factor

Table S4. Subgroup analyses of participants with prediabetes/diabetes and participants taking metformin intake *vs* on diet only

Outcome variable	Metformin (+) (n=18)			Metformin (-) (n=30)			Prediabetes (n=28)			Diabetes (n=20)		
	Placebo group (n=10)	Carnosine group (n=8)	*P*	Placebo group (n=14)	Carnosine group (n=16)	*P*	Placebo group (n=12)	Carnosine group (n=16)	*P*	Placebo group (n=12)	Carnosine group (n=8)	*P*
Change in adiponectin, [μg/ml]	-0.7±3.5 [a]	-2.1±3.4	0.40	-1.2±2.3	-1.05±3.2	0.88	-0.7±3.5	-2±2.7	0.29	-0.2±1.1	-0.2±3.9	0.45
Change in MCP-1, [pg/ml]	-11.7±47.2	-62.2±65.2	0.07	-33±65.4	-4.9±53.2	0.20	-2.1±48.2	-26.6±60.4	0.25	-46.2±61.2	-18.8±70.5	0.36
Change in CRP, [ng/ml]	-1.9 (-7, 6.7) [b]	1.6 (-9.2, 3.6)	0.86	0.6 (-4.9, 10.5)	1.8 (-2.3, 5.9)	0.66	-1.3 (-9.9, 2.6)	2.1 (-2.3, 3.4)	0.63	1.8 (-2.5, 13.5)	1.3 (-17.5, 6.7)	0.33
Change in complement Factor D/adipsin, [μg/ml]	0.2±1.1	-0.1±1.6	0.61	-0.1±1.05	-0.1±0.7	0.96	0.2±1.01	-0.4±0.7	0.10	-0.1±1.2	0.5±1.4	0.32
Change in leptin, [ng/ml]	-0.5 (-3.6, 1.7)	-4.8 (-11.8, -2.4)	0.10	-2.3 (-4.2, 2.7)	-2.7 (-3.6, 0.8)	0.85	-2.2 (-4.1, 5.9)	-3.1 (-3.6, 0.6)	0.17	-0.6 (-3.5, 1.2)	-4.8 (-9.3, 3)	0.34
Change in resistin, [ng/ml]	-1.02±2.7	-0.2±2.1	0.50	-0.4±0.7	-0.4±0.7	0.88	-0.6±2.3	-0.3±1.4	0.62	-0.7±1.2	-0.5±1.1	0.64
Change in serpin E1/PAI-1, [ng/ml]	-16.9±42.2	-21.8±46.6	0.81	-23.1±31.1	-12.4±34	0.37	-24.9±32.7	-21.9±31.7	0.80	-16.02±38.8	-2.7±47.8	0.50
Change in IL-6, [pg/ml]	-0.1±0.6	-0.1±1.8	0.99	0.1±0.5	-0.05±0.6	0.57	-0.1±0.5	-0.3±0.8	0.40	0.1±0.6	0.5±1.4	0.41
Change in IL-10, [pg/ml]	-0.1±0.3	0.04±0.2	0.44	-0.03±0.2	-0.02±0.2	0.93	-0.1±0.2	-0.05±0.2	0.31	0.05±0.3	0.1±0.1	0.66
Change in TNF-α, [pg/ml]	-0.1±1.1	0.2±1.2	0.54	-0.2±1.1	-0.1±1.5	0.84	0.2±0.8	-0.2±1.1	0.26	-0.5±1.2	0.5±1.9	0.16

[a] Data are expressed as mean ± SD for change scores. All analyses performed using independent t-test for differences between groups

[b] Median; IQR in in parentheses (all such values for nonnormally distributed variables). Nonnormally distributed variables were log transformed to the base 10 before analysis.

[*]Abbreviations: MCP-1: monocyte chemoattractant protein-1, CRP: c-reactive protein, PAI-1: *plasminogen activator inhibitor*-1, IL-6*: interleukins*-6, IL-10: *interleukins*-10, TNF-α: tumour necrosis factor-α
